# Comprehensive Characterization of Solution-Cast Pristine and Reduced Graphene Oxide Composite Polyvinylidene Fluoride Films for Sensory Applications

**DOI:** 10.3390/polym14132546

**Published:** 2022-06-22

**Authors:** Dane Hintermueller, Ravi Prakash

**Affiliations:** Department of Electronics Engineering, Carleton University, Ottawa, ON K1S 5B6, Canada; danehintermueller@cmail.carleton.ca

**Keywords:** piezoelectricity, polyvinylidene fluoride, atomic force microscopy, Mason model, reduced graphene oxide, polymer composites, sensors

## Abstract

Pristine and doped polyvinylidene fluoride (PVDF) are actively investigated for a broad range of applications in pressure sensing, energy harvesting, transducers, porous membranes, etc. There have been numerous reports on the improved piezoelectric and electric performance of PVDF-doped reduced graphene oxide (rGO) structures. However, the common in situ doping methods have proven to be expensive and less desirable. Furthermore, there is a lack of explicit extraction of the compression mode piezoelectric coefficient ($d_{33}$) in ex situ rGO doped PVDF composite films prepared using low-cost, solution-cast processes. In this work, we describe an optimal procedure for preparing high-quality pristine and nano-composite PVDF films using solution-casting and thermal poling. We then verify their electromechanical properties by rigorously characterizing β-phase concentration, crystallinity, piezoelectric coefficient, dielectric permittivity, and loss tangent. We also demonstrate a novel stationary atomic force microscope (AFM) technique designed to reduce non-piezoelectric influences on the extraction of $d_{33}$ in PVDF films. We then discuss the benefits of our $d_{33}$ measurements technique over commercially sourced piezometers and conventional piezoforce microscopy (PFM). Characterization outcomes from our in-house synthesized films demonstrate that the introduction of 0.3%w.t. rGO nanoparticles in a solution-cast only marginally changes the β-phase concentration from 83.7% to 81.7% and decreases the crystallinity from 42.4% to 37.3%, whereas doping increases the piezoelectric coefficient by 28% from $d_{33}$ = 45 pm/V to $d_{33}$ = 58 pm/V, while also improving the dielectric by 28%. The piezoelectric coefficients of our films were generally higher but comparable to other in situ prepared PVDF/rGO composite films, while the dielectric permittivity and β-phase concentrations were found to be lower.

## 1. Introduction

Semi-crystalline piezoelectric fluoropolymers, such as polyvinylidene fluoride (PVDF), are widely explored materials that can be synthesized as films, membranes, and fibers for a wide range of applications [1], including energy harvesting [2], piezoelectric actuators [3], ultrasonic transducers [4], porous membranes [5], and pressure sensors [6,7,8], to list a few. These devices are commonly fabricated using techniques, such as solution-casting [9], spin coating [10], electrospinning [11], 3D printing [12], electrospray deposition [13], and spray coating [14]. PVDF and co-polymer structures offer advantages, such as low cost, facile manufacturing, high mechanical strength, chemical stability, flexibility, good piezoelectricity, and biocompatibility [15]. However, when compared to ceramic piezoelectrics, PVDF suffers from a low electromechanical coupling coefficient, dielectric constant, and piezoelectric coefficient [15], limiting their compressive-sensing capacity to higher pressures on the order of kilopascals [16].

As interest in wearable technologies increases, parameters, such as flexibility, biocompatibility, and mechanical/chemical stability, have become highly desirable for tissue-interfaced healthcare devices requiring pressure data, such as tactile sensors, arterial pulse sensors, and intracranial sensors, among others [17]. PVDF-based structures, therefore, pose an excellent material for use in such systems. One example is Persano et al.’s self-powered tactile pressure sensors composed of aligned PVDF-trifluoroethylene (TrFE) nanofiber with very high sensitivity [18]. Another based on a more conventional piezoresistive architecture is a flexible tactile sensor using a promising polymeric composite, Velostat^®^ (Desco Industries Inc., Chino, CA, USA) [19]. Generally, sensitivity is one of the most important and commonly reported parameters for pressure sensors [17]. Unfortunately, pure PVDF films have intrinsically low compressive sensitivities; therefore, techniques to enhance its piezoelectric coefficient while maintaining flexibility and biocompatibility are being actively investigated [15].

The pressure-sensing capabilities of PVDF is correlated with the degree of crystallinity, the phase composition of the crystallites, and their orientation [20]. In general, PVDF crystalizes into five main phases: α,β,γ, δ, and ε of which β, γ, and δ are electroactive with the β-phase possessing the strongest dipole moment and best piezoelectric, pyroelectric, and ferroelectric properties [15]. The crystalline phases can be formed in different proportions based on the fabrication techniques with most works aimed at inducing β-phase dominant films [15]. The β-phase for PVDF films has been commonly produced using mechanical stretching [21], from a melt under high pressure [22,23], through quenching at low temperatures [24], solution-evaporation below 70 °C in a solution-cast [9], and via both organic and inorganic doping [25]. Carbon-based materials, such as graphene with their exceptional mechanical, thermal, and electrical physical properties, have shown themselves as ideal fillers to improve the intrinsic properties of polymers while maintaining flexibility and biocompatibility at low concentrations [26] with numerous uses in sensing applications [27,28,29]. The solution-cast technique with graphene filler has demonstrated exceptional advantages over other methods due to its scalability, repeatability, and simplicity [30]. Moreover, as summarized in the review paper [31], various articles have demonstrated that doping PVDF with small concentrations (<1%w.t.) of reduced graphene oxide (rGO) has improved dielectric permittivity, increased electroactive β-phase concentration, and enhanced pressure-induced output voltages. However, few studies explicitly quantify the sensitivity of these composite films by determining the compressive-mode piezoelectric coefficient ($d_{33}$) [31]. Recent works which experimentally extract the coefficient have prepared the composite films (optimally with 0.3%w.t. rGO) via in situ chemical or thermal reduction of graphene oxide (GO) [32,33,34,35], rather than dispersing rGO into PVDF solutions using solution-cast techniques. However, in situ reduction is complex, expensive, and poorly controlled [34]. We, therefore, aim to determine if the latter, i.e., solution-cast rGO filler suspension method can produce PVDF/rGO (0.3%w.t.) composites with comparable enhancements in properties to those employing in situ reduction of graphene oxide [33,34,35]. We accomplish this by fabricating free-standing solution-cast PVDF/rGO composite films enhanced using thermal pressing and electrical poling and subsequently characterize the chemical (β-phase concentration and crystallinity), electromechanical ($d_{33}$), and electrical (permittivity and loss tangent) properties of these composites. The chemical properties were determined using Fourier-transformed infrared spectroscopy (FTIR), X-ray diffraction crystallography (XRD), and differential scanning calorimetry (DSC), where only by combining these three techniques can one obtain unambiguous film characterization [36].

Most commonly, the piezoelectric coefficient of PVDF-based devices is determined using commercially available piezometers [37] or via an atomic force microscope (AFM)-based technique known as piezoforce microscopy (PFM) [38], each approach having its advantages and disadvantages. Piezometers typically employ the “Berlincourt method” which is based on a basic operating principle, whereby a known small oscillating force is applied to the sample, and the charge generated due to the direct piezoelectric effect is measured [37]. Although simple without any established design standards for this technique, there remain systematic differences between various commercial apparatuses leading to a loss of confidence in $d_{33}$ measurements reported in the literature [39]. In addition, the clamping forces needed to keep the sample in place have been observed to particularly affect measured results for soft piezoelectrics, such as PVDF [39]. The latter technique involves scanning the sample surface with a conductive AFM probe and measuring the localized surface strain induced by a tip-applied ac voltage using a lock-in amplifier [38]. This technique has benefited from exceptionally high resolution and ease of use [40]. However, the electromechanical response measured in PFM is caused by the piezoelectric effect superimposed onto the influence of electrochemical strain, electrostriction, and electrostatic effects, among others [41]; these effects can not only reduce the accuracy of PFM measurements but if not considered carefully can dominate the observed response [41]. Considering these pitfalls, we propose an improved method of extracting $d_{33}$ in PVDF films by analysing the AFM cantilever dynamics and designing the measurement to minimize the non-piezoelectric influences present in conventional PFM.

## 2. Materials and Method

### 2.1. PVDF Film Preparation

Four different types of solution-cast PVDF structures were prepared in this study: two pure PVDF films and two 0.3%w.t. rGO doped PVDF (PVDF/rGO) films. One pure and one rGO doped film were subjected to a mechanical hot press and thermally poled, while the other two were examined as cast. The films were prepared by dissolving PVDF pellets (Sigma Aldrich, Burlington, MA, USA) in N, N-dimethylformamide (DMF) to form a solution with a 20%w.t. concentration. The solution was continuously stirred at 200 rpm in a hermetically sealed bottle using a magnetic stirring rod for 2 h at 60 °C. The 0.3%w.t. rGO-doped PVDF films [33,34,35] were created by separately dispersing powdered rGO (KLG-RGO-C; Kennedy Labs, Canada) in DMF and mixing the solution in an ultrasonic bath for 2 h at 60 °C to obtain a well-dispersed mixture. The rGO powder used in this study was produced by reducing graphene oxide employing the modified Hummer’s method and was specified to contain a nominal carbon content of 85%. Subsequently, the 20%w.t. PVDF in DMF solution was combined with the rGO in DMF mixture and stirred at 60 °C for 1.5 h to ensure homogeneity. The solutions were then poured onto a 60 °C pre-heated aluminum surface and smoothed to the appropriate height to produce ~120 μm thick dry films using an automatic film applicator (TQC sheen, Capelle aan den Ijssel, The Netherlands). The wet films were evaporated at 60 °C [9] in an oven for 3 h, removed, and cooled to room temperature in the open air. Next, a metal vice was placed in the oven and heated to 140 °C. One pure and one rGO-doped film were manually hot-pressed between the vice clamps and then placed back in the oven for 10 min at 140 °C [21]. After this hot-pressing procedure, the films were rapidly cooled by removing the samples from the oven and quenching in chilled 10 °C deionized water. Finally, the top and bottom electrodes composed of 50 nm/125 nm chromium/gold were deposited through a shadow mask using the Angstrom Covap physical vapor deposition system to produce a pair of active capacitor areas of 3 mm × 3 mm for each film structure.

**Figure 1:**
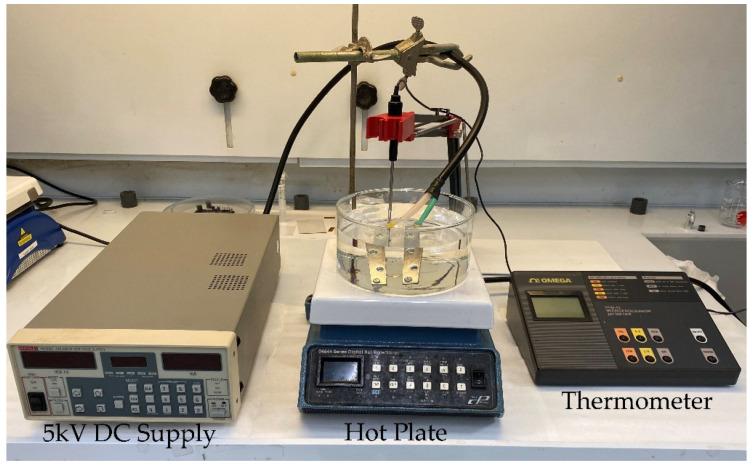
Thermal poling apparatus.

Thereafter, the hot-pressed PVDF and PVDF/rGO films were poled at 90 °C [42] in a heavy mineral oil bath using a 5 kV DC supply (Keithley 248), as seen in Figure 1. The voltage was ramped up to 5 kV in 10 V steps over a 5-min interval and then poled for 2 h at 5 kV (~40 MV/m). The sample electrodes were electrically connected to the voltage supply using steel metal brackets fixed on glass slides, ensuring that the freestanding films remained flat during poling. The samples were subsequently cooled to room temperature while the electric field remained applied to permanently fix the dipoles along the poling direction. Finally, the samples were cleaned with isopropyl alcohol, and their electrodes were connected in a short circuit for 12 h before taking measurements to neutralize any remnant charges. The unpressed PVDF and PVDF/rGO samples were observed to exhibit dielectric breakdown after 15 min of thermal poling. Therefore, the unpressed samples were not subjected to thermal poling in this study. This breakdown was attributed to the high porosity and surface roughness of the films, as will be discussed in Section 3.1.

### 2.2. Methods Used for Film Characterization

#### 2.2.1. Chemical Characterization Using FTIR-ATR, XRD, and DSC

As a semi-crystalline polymer that crystalizes into multiple different phases, a combination of several characterization techniques is required to provide a confident interpretation of PVDF film composition. FTIR and XRD together can be used to extract the proportion of crystalline phases present with the structure, while DSC can be used to quantify the crystallinity of the film [36]. It has been reported that the α-phase infrared spectrum exhibits CF_2_ and skeletal-bending peaks at 612 and 764 cm^−1^, CH_2_-rocking peaks at 795 cm^−1^, and CH_2_-twisting peaks at 795 cm^−1^; the β-phase exhibits a CH_2_-rocking peak at 840 cm^−1^ and another at 1274 cm^−1^ [43]. Under the assumption of minimal γ and δ phases, the relative proportion of β-phase can be determined based on the Lambert–Beer law from the absorbance at the α and β-phase peaks found at 764 cm^−1^ and 840 cm^−1^ [43], respectively. The β-phase concentration of the PVDF films was estimated using the Equation (1) [43],

(1) $$\begin{array}{c} F\left(\beta \right)=\frac{A_{\beta }}{\left(\frac{K_{\beta }}{K_{\alpha }}\right)A_{\alpha }+A_{\beta }},\; \end{array}$$

where $A_{\alpha }$ and $A_{\beta }$ are the absorbances at α (764 cm^−1^) and β (840 cm^−1^) absorbance peaks, respectively, and $K_{\alpha }\;$= 6.1 × 10^4^ and $K_{\beta }\;$= 7.7 × 10^4^ cm^2^/mol are the absorption coefficients at their respective wavenumbers. The absorption spectrum was collected in attenuated total reflection (FTIR-ATR) mode with a resolution of 0.483 cm^−1^ using an FTIR spectrometer (Thermo Scientific Nicolet iS50, Waltham, MA, USA) by averaging 16 automatic-baseline-corrected scans.

The characteristic X-ray diffraction peaks angles for the respective crystal planes used to identify the crystallites in PVDF have been reported for the α-phase as 17.66° (100), 18.30° (020), 19.90° (110), and 26.56° (021); for the γ-phase as 18.50° (020), 19.20° (002), and 20.04° (110); and for the β-phase as 20.26° (110/200) [36]. The X-ray diffraction measurements were performed using a Rigaku Ultima IV powder diffractometer with an X-ray source of Cu Kα (λ = 1.5418 Å) within the range of 10° < 2 < 30° at a scan rate of 0.5°/min.

DSC provides information on the thermal behavior of PVDF under phase transformations by measuring the heat flow through the sample through progressive heating. The area under the exothermic heat flow curve as a function of time through the melting point was used to determine the degree of crystallinity in the samples via [44]

(2) $$\begin{array}{c} X_{c}\left(\%\right)=\frac{\Delta H_{m}^{sample}}{\left(1-\phi \right)\Delta H_{m}^{pure}}, \end{array}$$

where $\Delta H_{m}^{sample}$ is the area under the melting curve, $\Delta H_{m}^{pure}$ is heat enthalpy of pure 100% crystalline PVDF of 104.50 Jg^−1^, and ϕ is the weight percentage of rGO filler. Samples of ~2 mg were analyzed using a differential-scanning calorimeter (TA Instruments Q100, Bellingham, WA, USA) at a heating rate of 5 °C/min from room temperature to 200 °C.

#### 2.2.2. Electromechanical Characterization Using Static AFM

The electric displacement ($D_{m}$) and strain ($\varepsilon_{ij}$) tensors of piezoelectric materials are expressed as [19]

(3) $$\begin{array}{c} D_{m}=d_{mkl}\sigma_{kl}+\epsilon_{mn}E_{n},\; \end{array}$$

(4) $$\begin{array}{c} \varepsilon_{ij}=S_{ijkl}\sigma_{kl}+d_{nij}E_{n}, \end{array}$$

where $\epsilon_{mn}$ is the stress-free permittivity,$\;E_{n}$ is the electric field strength, $d_{mkl}$ and $d_{nij}$ are the piezoelectric coefficients, $\sigma_{kl}$ is the mechanical stress, and $S_{ijkl}$ is the short-circuit mechanical compliance. Since our PVDF films are free standing, no stress component is assumed. In addition, we aim to measure the compression mode (33) coefficient where the voltage and displacement are applied and recorded across the sample thickness (3). Therefore, we can express the total strain along the thickness by the sum

(5) $$\begin{array}{c} \varepsilon_{33}=d_{31}E_{3}+d_{32}E_{3}+d_{33}E_{3},\; \end{array}$$

where the electric field strength and strain are E=ΔV/t and ε=Δt/t , respectively. Thus, if we ignore the in-plane influences of $d_{31}$ and $d_{32}$ [45], the piezoelectric coefficient can be approximated as

(6) $$\begin{array}{c} d_{33}=\frac{\Delta t}{\Delta V} \end{array}$$

with Δ*t* being the change in sample thickness and ΔV the voltage across the sample.

**Figure 2:**
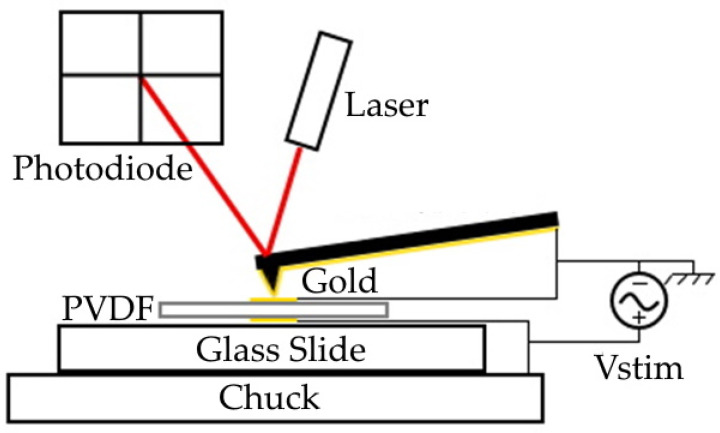
AFM configuration for piezoelectric coefficient measurement.

A schematic of the measurement apparatus used to extract the piezoelectric coefficient taken at ambient room temperature is depicted in Figure 2. We first set the AFM (Veeco Dimensions 3100, Cambridge, MA, USA) in contact mode at the center of the sample electrode and configured it to measure cantilever deflection at a static location. We then confirmed that the AFM tip and sample electrode were electrically connected (at GND) and supplied a 0.5 Hz AC positive voltage to the bottom electrode for several voltages between 10 V and 70 V. The piezoelectric coefficient was subsequently determined by first averaging three separate measurements of the peak-to-peak cantilever deflections versus driving voltage and then estimating $d_{33}$ from the linear slope [45,46]. The AC signal was supplied by using the analog control of the high voltage DC supply. This offered advantages of internal current limiting operation and precluded the necessity of a high-voltage amplifier often required to obtain reasonable signal-to-noise ratios when characterizing low piezoelectric coefficient materials. Since solution-cast PVDF films do not preferentially align dipoles along a common axis without poling [47], the piezoelectric coefficient of the unpressed PVDF and PVDF/rGO samples was not measured in this study.

Regarding techniques used to enhance the accuracy of piezoelectric coefficient measurements, recall that the measured cantilever deflection is due to the superposition of piezoelectricity, electrochemical strain, electrostriction, and electrostatic influences [41]. Electrochemical or Vegar strain is the surface displacement induced by diffusion, electromigration, or chemical reactions of mobile ions caused by an electric field [41]. PVDF has very low ionic conductivities [48]; therefore, the influence of this effect on the measurements is expected to be very small. It has even been confirmed that amorphous PVDF does not exhibit noticeable electrochemical strain [49] with crystalline PVDF suspected to behave similarly. Moreover, the electrostrictive effect of PVDF has been shown to produce strains of ~1% at high-electric-field strengths (10 MV/m) [50]. This effect would be negligible in our setup due to the presence of the top electrode which ensures a uniformly distributed electric field through the thickness. However, this influence may be significant in traditional PFM measurements because the sharp conductive tip produces high local electric field strengths near the contact point. Finally, the electrostatic effect can be illuminated by the force experienced by an AFM cantilever given as [51]

(7) $$\begin{array}{c} F_{es}=-\frac{1}{2}\frac{dC}{dz}\left(U-\Psi \right)+\sum \frac{q_{t}q_{s}}{4\pi \epsilon_{o}z^{2}}\; \end{array}$$

The variable dC/dz is the positionally dependent probe sample capacitance which depends on the geometry of the tip and sample, U is the difference in potential energy between the tip and sample, Ψ is the difference in work function between the tip and sample, and $q_{t}$ and $q_{s}$ are the trapped charges on the tip and sample, respectively. The magnitude of the cantilever displacement due to this electrostatic interaction can consequently be expressed using the spring-mass force model (F=kz) and Equation (7) as

(8) $$\begin{array}{c} z_{es}=k^{-1}F_{es}=k^{-1}\left(-\frac{1}{2}\frac{dC}{dz}\left(U-\Psi \right)+\sum \frac{q_{t}q_{s}}{4\pi \epsilon_{o}z^{2}}\right) \end{array}$$

It is apparent from Equation (8) that the electrostatic influence can be minimized by having the sample surface and tip at the same potential, coating the two surfaces with the same material, reducing the effective influence of any surface charges, and increasing the cantilever spring constant. Our AFM measurement apparatus produces such a system through the sample’s gold top electrode and deposition of 5 nm/60 nm Cr/Au onto the tip-side of a Pyrex-Nitride (PNP-DB; Nano World, Neuchâtel, Switzerland) rectangular AFM cantilever with a sufficiently large stiffness [52] of 0.4 N/m. In contrast, the sample surface in PFM is typically not the same material as the tip, and charge injection during scanning can serve to increase the electrostatic interaction [41].

#### 2.2.3. Electrical Characterization Using the Mason Model

**Figure 3:**
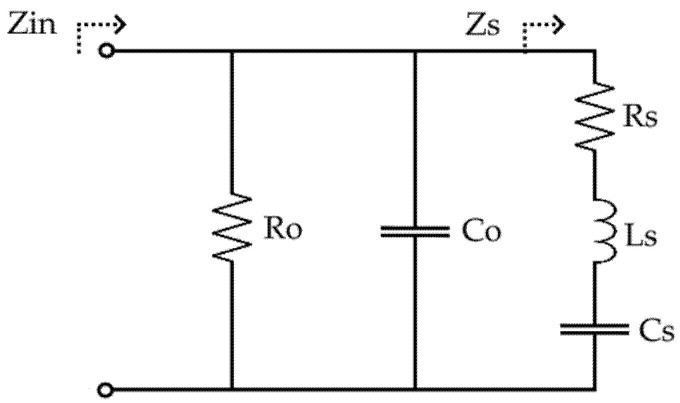
Mason model equivalent circuit for PVDF.

The Mason model is an equivalent circuit used to model the electromechanical properties of lossy piezoelectric resonator materials, such as PVDF, requiring only impedance and film dimensions as inputs [53]. This model is especially useful for polymeric materials with high dielectric and mechanical losses, coupled with high frequency and temperature-dependent dielectric, elastic, and piezoelectric properties [54]. In this model, dielectric losses are accounted for by a shunt loss resistor (Ro) and a bulk film capacitor (Co), as shown in Figure 3 [54]. The equivalent mechanical impedance is represented by the series RLC circuit (Zs) with the mechanical losses accounted for by the loss resistor (Rs) [54].

When the stimulation frequency is far from thickness resonance at ~10 MHz ($f_{o}\sim \frac{c}{2t}$, where c is the speed of sound in PVDF [55]), the influence of the series RLC circuit becomes negligible, and $Z_{s}$ approaches zero. Therefore, the dielectric loss tangent and relative permittivity can be expressed respectively as [54]

(9) $$tan\delta_{e}=-\frac{1}{tan\left(\theta_{z}\right)}$$

(10) $$\epsilon_{33}=\frac{t}{\left|Z_{in}\right|\omega A\sqrt{\left(\;\tan^{2}\delta_{e}+1\right)}\;}$$

where $\left|Z_{in}\right|$ and $\theta_{z}$ are the input impedance magnitude and phase, A is the active capacitor area, and t is the film thickness. The electrical impedance was measured at ambient room temperature using the Agilent 4294A precision impedance analyzer (Agilent Technologies Inc., Santa Clara, USA), between 1 kHz and 5 MHz. The impedance of the electrodes was then subtracted from the total sample impedance to isolate the intrinsic properties of the polymer.

## 3. Results

### 3.1. Physical Characteristics

**Figure 4:**
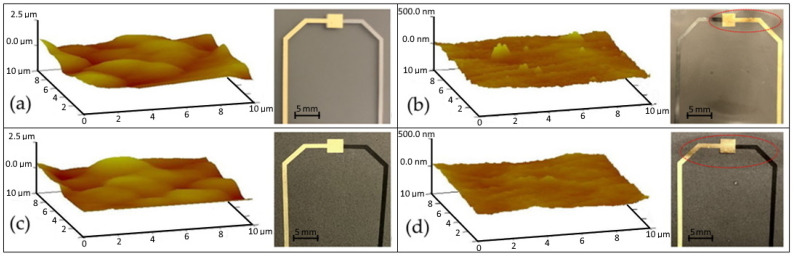
PVDF films and their surface topology: (**a**) pristine unpressed; (**b**) pristine hot pressed; (**c**) rGO doped unpressed; (**d**) rGO doped hot-pressed films. Note the hot-press-induced optical transparency annotated in red for (**b**,**d**).

The surface morphology for all four samples over an area of 100 μm^2^ can be seen in Figure 4. The root-mean-squared roughness of the unpressed PVDF and PVDF/rGO samples were determined to be 277 nm and 271 nm, respectively. In contrast, the hot-pressed PVDF and PVDF/rGO exhibited a 10-fold reduction in roughness at 29.3 nm and 22.2 nm, respectively. The high-surface roughness and porosity of the unpressed solution-cast films were observed to render them prone to dielectric breakdown during poling [21]; hot pressing was observed to prevent this breakdown. Although the manual hot-press method is demonstrated as an effective low-cost technique to reduce porosity in small areas, it is limited in producing uniform pressure across the whole film surface. This can be observed in Figure 4b,d, where only a small, targeted film area near the metal electrodes highlighted in red exhibited hot-press-induced optical transparency.

### 3.2. Chemical Characteristics

**Figure 5:**
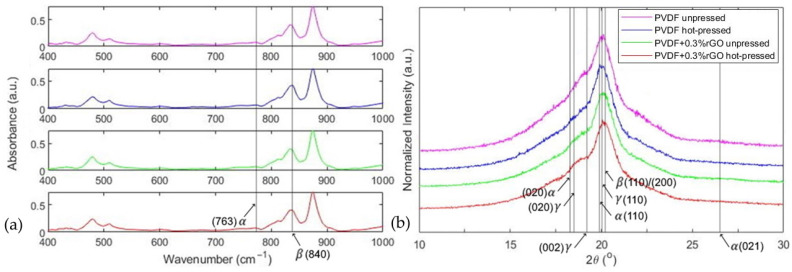
(**a**) FTIR-ATR absorbance spectrums; (**b**) X-ray diffraction spectrums.

**Figure 6:**
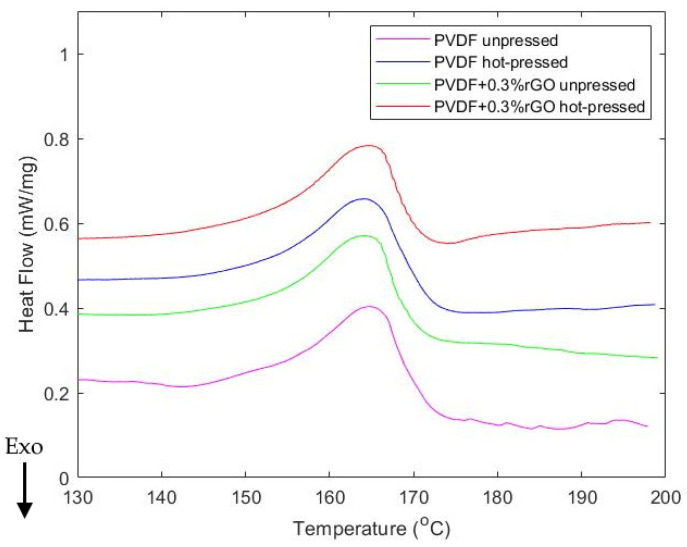
DSC thermogram melting curves.

The FTIR-ATR absorbance and normalized XRD spectrums used to confirm the crystalline phase information of the samples are given in Figure 5a,b, respectively. The relevant α (764 cm^−1^) and β (840 cm^−1^) FTIR absorbance peaks and phase-dependent crystal diffraction planes in the XRD spectrums are also annotated in Figure 5. Moreover, the DSC melting enthalpy curves are depicted in Figure 6. The parameters extracted from FTIR and DSC are summarized in Table 1; these parameters include the β-phase concentration obtained using Equation (1), the crystallinity obtained using Equation (2), and the melting temperature determined from the temperature at which maximum heat flow is observed within DSC curves.

### 3.3. Electromechancial Characteristics

**Figure 7:**
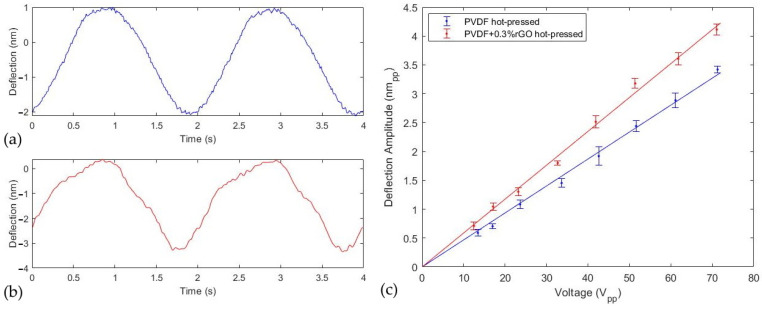
Deflection of AFM cantilever under 60 Vpp at 0.5 Hz for (**a**) PVDF; (**b**) PVDF + 0.3%w.t. rGO; (**c**) deflection amplitude of AFM cantilever for stimulation between 10 and 70 Vpp.

The plots in Figure 7a,b demonstrate the time-dependent surface deformation of the PVDF and PVDF/rGO samples based on the AFM cantilever deflection for ~60 Vpp AC stimulation at 0.5 Hz. As predicted by Equation (6), a linear plot of deflection amplitude as a function of stimulation voltage between 10 Vpp and 70 Vpp was observed in Figure 7c. The least squares fit of the displacement versus applied voltage slope was used to extract the piezoelectric coefficient of the PVDF and PVDF/rGO films and their magnitudes were calculated to increase by 28% as $d_{33}\;$= 45 pm/V and $d_{33}\;$= 58 pm/V, respectively. To confirm no electrostatic interaction between the tip and top electrode we applied 20 Vpp to the sample/tip and measured the cantilever deflection with the tip positioned over the top electrode but outside the overlapping active capacitor region; no change in deflection signal was detected with both the tip above the surface and in contact.

### 3.4. Electrical Characteristics

**Figure 8:**
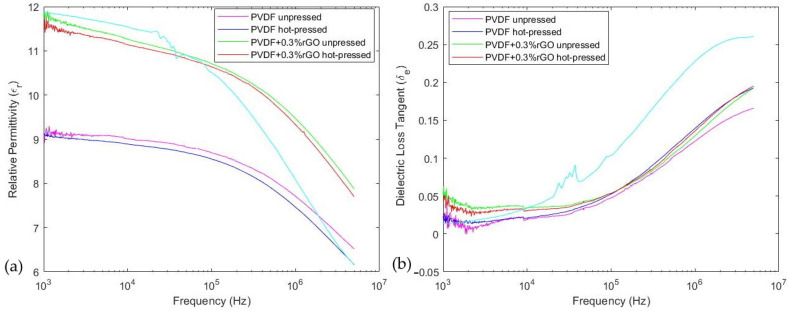
Micrographs showing frequency sweeps of (**a**) relative permittivity, and (**b**) dielectric loss tangent of PVDF samples.

The relative permittivity and dielectric loss tangent were calculated using Equations (9) and (10) and are depicted in Figure 8a,b, respectively. The variation in permittivity and loss tangent through the introduction of dopants and hot pressing is discussed in detail in Section 4.3. Moreover, the magnitudes of these parameters at 1 kHz are summarized in Table 2. We also included the electrical parameters of a commercial PVDF film (TE Connectivity, Troy, MI, USA) which had an active area of 8 cm^2^, a thickness comparable to our films (~110 μm), and 5 μm thick silver ink electrodes to establish an external comparison.

## 4. Discussion

### 4.1. Chemical Characterization

The FTIR results outlined in Table 1 indicated an enhancement in β-phase concentration through hot pressing from 80.1% to 83.7% for the PVDF films and from 80.8% to 81.7% for the PVDF/rGO films, respectively. The mechanism for improvement due to the hot press can be attributed to the increase in chain mobility with the application of unidirectional force at high temperatures, inducing dipole rotation around the chain axis from the α-phase into the β-phase configuration [56]. We also noted that the enhancement in β-phase between the pressed and unpressed samples may have been due to the rapid cooling with chilled water, which has been shown to increase β-phase concentration [22,24]. The slight decrease in β-phase concentration observed when comparing pressed PVDF and PVDF/rGO samples demonstrated that the increase of β-phase to 100% observed from in situ reduction [33,34,35] was not paralleled in the microparticle suspension technique. This is suspected to be caused by the re-agglomeration of the graphene sheets due to their strong intramolecular van der Waals forces during film evaporation, leading to low dispersion of graphene in the polymer minimizing their interaction [57]. Therefore, future studies should be directed toward techniques to prevent the re-agglomeration of rGO in particle suspension techniques. However, in agreement with this study, the introduction of rGO between 0–1.6 vol% in solution cast PVDF films prepared via microparticle suspension has lowered the β-phase concentration between 82.15–87.30% compared to pristine PVDF as 91.02% [58].

Furthermore, the XRD intensity curves depicted in Figure 7b support the predominance of the β-phase in all samples, as suggested by the FTIR data. This is demonstrated through the presence of a singular dominant peak centered around 20.3° in all samples which have been attributed to the (110/200) crystal planes of the β-phase [59,60,61,62]. As seen in Figure 7b, the (110) α and γ crystal diffraction angle planes are very close to the β-phase diffraction angle, implying that the observed intensity spectrum is a superposition of the diffraction intensities originating from all three phases over this region. Therefore, to further distinguish the three phases, we may infer β-phase presence by observing only a very small-intensity peak for all samples around the (020) α and γ planes in the 18.5° region and no peak for the (021) α and (002) γ planes near the 26.5° and 19.2° regions, respectively. We also notice that the pristine hot-pressed PVDF film exhibited the smallest peak in the 18.5° region of all samples, thereby validating the FTIR results which quantify the hot-pressed PVDF pure film as having the highest β-phase concentration. It is also worth mentioning that XRD can also be used to quantify crystalline proportions in PVDF. For example, with films prepared under similar casting conditions, Gradys et al. [63] has reported comparable β-phase concentrations using wide-angle X-ray diffraction. However, extracting the crystalline ratios using XRD is very involved and requires fitting over 20 parameters [63], with FTIR clearly being a much simpler technique.

Finally, DSC was also used to provide further insight into both the phase composition of the films and the effects of small concentrations of rGO dopants on crystallinity, as seen in Figure 8 and Table 2. Generally, each crystalline phase of PVDF has a different melting curve and peak temperature with the measured response manifesting as the proportional sum of the melting curves of each phase [36]. The β-phase and α-phase are difficult to discern from one another from the enthalpy curves since they have significant overlap with peak temperatures found in the 165–172 °C range, while the γ-phase has a narrow peak between 179–180 °C or 189–190 °C depending on synthesis techniques [36]. In Figure 6 all samples exhibit a broad peak at ~165 °C which coincides well with the expected curve of β/α -phase films. As seen in Table 2, we also notice a decrease in crystallinity between the pure PVDF samples and the rGO-doped samples within the range of 37–42% which has often been reported for PVDF graphene-based composites [62,64,65]. This reduction has been associated with different features, such as the length, curvature, and distribution of the dopant which can interrupt the packing of polymer chains into crystallites [66]. As done in this study and most DSC analyses of PVDF, the heat enthalpy of 100% crystalline PVDF was taken to be 104.50 Jg^−1^. However, it is worth noting that this value was determined by Nakagawa et al. [67] for α-phase dominant samples, whereas recently, the pure heat enthalpy of β-phase PVDF has been reported as 219.7 Jg^−1^ [63]. Therefore, to enhance the accuracy of crystallinity calculations for multiphase PVDF, future studies can be directed towards developing a weighted pure heat enthalpy magnitude based on the quantitative phase proportions determined by FTIR.

### 4.2. Electromechanical Characterization

The results presented in Section 3.3 demonstrated the successful enhancement of the piezoelectric coefficient via the rGO micro-particle suspension technique while ensuring no electrostatic interaction between the sample and tip. Table 3 depicts a summary of the piezoelectric coefficient and β-phase concentration of PVDF films prepared under similar conditions. It is apparent from Table 3 that the measured piezoelectric coefficients seem to have some systematic differences based on the measurement techniques. We notice that the piezometers record the lowest piezoelectric measurements which may be due to the softness of the material and the subsequently enhanced clamping force effects [39]. Studies that use similar static AFM techniques to ours report higher piezoelectric coefficients than through piezometers, with the highest reports from PFM measurements. The enhanced piezoelectric coefficient observed in this study can be associated with the processing conditions, high β-phase concentration, the free-standing nature of the measurement, and the steps taken to minimize electrostatic interaction. Since the sample surface is free to deflect freely along the (3) direction without any backing-induced compliance, an increase in effective piezoelectric coefficient can be expected over other studies [52]. The PFM technique neglects the electrostatic influence caused by the work function difference between sample and tip which can contribute to a systematic increase in an observed piezoelectric effect. In addition, very often with PFM measurements, the ac voltage is applied at a frequency near the contact resonance of the tip [68] which magnifies the cantilever deflection amplitude via $A=d_{33}V_{ac}Q$, leading to overestimations in measured response either due to ignoring the quality factor (ranging from 10–100) or its inaccurate estimation [69]. Additional variability seen in Table 3 can be attributed to differences in fabrication procedures, such as evaporation temperatures, poling fields, mechanical stretching, pressing conditions, and dopant concentrations which influence piezoelectric properties and β-phase concentration [15]. Our rGO-doped sample was observed to have improved the piezoelectric coefficient of reasonable comparability with other reports. The improved piezoelectric response in our case was not correlated with the β-phase concentration similarly to Pariy et al. [32]. Instead, the foundational increase has been associated with the charge mobilization in the composite which enhances heteropolarization by the interaction of the rGO π-electrons and the CH_2_ carbonyl subgroups in PVDF [33].

It is relevant to report that the piezoelectric coefficient determined by the procedure described in Section 2.2.2 only quantifies the coefficient for a localized region of the sample since the radius of the AFM tip is on the order of 10 nm. Therefore, measurements can vary based on local crystallinity, surface morphology, and distance from the edge of the active region. Thus, it is suggested to make measurements on a smooth sample near the center of the active region to minimize the influence of local surface peaks and compliance induced by the PVDF outside the electrode boundary [37]. It is also notable that some limitations arise in this method since the deflection sensitivity of the AFM cantilever must be calibrated to determine the change in photodiode signal as a function of cantilever deflection which has been reported to contribute up to 10% uncertainty [52]. Moreover, the AFM should be configured with low-contact forces due to the low-elastic modulus of PVDF. Otherwise, as the surface oscillates vertically the tip can indent the sample, reducing the observed deformation. This is accomplished by modulating the deflection setpoint and minimizing the feedback gains while ensuring sustained contact.

### 4.3. Electrical Characterization

The relative permittivity depicted in Figure 8a and Table 2 demonstrated that the commercial PVDF film had the highest permittivity at low frequencies, with the rGO-doped samples slightly lower and pristine PVDF at the lowest. We also noted that hot pressing was observed to slightly decrease the dielectric permittivity and loss tangent which can be attributed to the increase in crystal-packing density, limiting free electric dipole movement [70]. Through the introduction of 0.3%w.t. rGO, we observed an increase in permittivity of 28% from 9.01 to 11.52 at 1 kHz. This amelioration in dielectric permittivity at frequencies below 100 kHz can be mainly attributed to the Maxwell–Wagner–Sillars (MWS) interfacial polarization caused by the heterogeneous conductivity of rGO and PVDF [52]. The MWS effect is produced by the entrapment of free charges between the thin insulator (PVDF) and conductor (rGO) interfaces producing many micro-capacitor structures [71]. The increased charge mobility caused by heterogeneous conductivity also led to the measured increase in loss tangent for the rGO-doped films [71]. The improvements in permittivity from rGO doping are comparable but lower than other solution-cast rGO-composite films, such as the ~57% increase from 7 to 11 observed with 0.35%w.t. [72] and the ~88% increase from 9 to 17 with 0.39%vol [71]. However, these enhancements were much smaller than those arising from in situ reduction, such as the ~400% increase from ~17 to ~70 at 1 kHz with 0.3%w.t. rGO reported by Rahman et al. [33]. This discrepancy can be understood because in situ reduction is known to enhance the polymer–graphene interaction significantly while ensuring homogeneous dispersion compared to the simpler solution method [30].

## 5. Conclusions

In this study, we have produced low-cost PVDF/rGO-composite films using the solution method and characterized them using a robust combination of FTIR-ATR, XRD, DSC, AFM, and electrical techniques. AFM was uniquely configured to experimentally measure the piezoelectric sensitivity of hot-pressed and thermally poled PVDF and PVDF/rGO composites with an emphasis on techniques for reducing the electrostatic influences typically present in PFM. The piezoelectric coefficient for PVDF and 0.3%w.t. doped rGO films measured using our technique was determined to be $d_{33}\;$= 45 pm/V and $d_{33}\;$= 58 pm/V, respectively. These high compressive sensitivities help position PVDF/rGO composite films as useful bio-integrated pressure-sensing devices. The overall increase in $d_{33}$ was discussed in terms of film-processing conditions and systematic differences between our technique, piezometer-based measurements, and piezoforce microscopy. The chemical and electrical results indicated that 0.3%w.t. rGO doping in hot-pressed PVDF decreased the β-phase concentration slightly from 83.7% to 81.7%, decreased the crystallinity from 42.4% to 37.3%, and increased dielectric permittivity by 28%. We conclude that the solution-cast rGO filler suspension method can produce composites with comparable piezoelectric enhancements and crystallinity to those employing in situ reduction but not with as impressive of improvements in permittivity and β-phase concentration. Future studies directed at techniques to prevent re-agglomeration of rGO in solution-cast methods would be beneficial to provide an enhanced polymer–graphene interaction which can improve both permittivity and β-phase concentration. The fabrication and characterization techniques explored in this study will be valuable for researchers interested in enhancing the sensitivity of low-cost flexible piezoelectric sensors.

## Figures and Tables

**Table 1:** Compiled chemical characteristics of the four solution-cast PVDF films.

Sample	β-Phase Fraction (%)	Crystallinity (%)	Melt Temperature (°C)
PVDF unpressed	80.1	39.4	165.0
PVDF hot-pressed	83.7	42.4	164.3
0.3%w.t. rGO doped and unpressed	80.8	37.2	164.5
0.3%w.t. rGO doped and hot-pressed	81.7	37.3	164.5

**Table 2:** Extracted dielectric attributes of PVDF composites.

Sample	Relative Permittivity @ 1 kHz	Loss Tangent @ 1 kHz
PVDF unpressed	9.15	0.025
PVDF hot pressed	9.01	0.017
0.3%w.t. rGO doped and unpressed	11.70	0.053
0.3%w.t. rGO doped and hot pressed	11.52	0.045
PVDF commercial film	11.87	0.013

**Table 3:** Comparative literature review of PVDF piezoelectric coefficient measurements.

Fabrication Procedure	F(β)%	$\left|d_{33}\right|(\mathrm{pm}/V)$	Technique	Source
PVDF solution cast, hot pressed, thermally poled	83.67	45	Static AFM	Current study
PVDF + 0.3%w.t. rGO solution-cast, hot pressed, thermally poled	81.69	58	Static AFM	Current Study
PVDF solution-cast, poled	100	22	Piezometer	[34]
PVDF + 0.3%rGO in situ, solution-cast, poled	~100	37	Piezometer	[34]
PVDF poled, solution cast	2	13	Piezometer	[35]
PVDF + 0.25%w.t. rGO in situ, solution-cast	37	25	Piezometer	[35]
PVDF spin coated, thermally poled	80	20	Piezometer	[10]
PVDF spin coated, stretched, poled	75	37	Static AFM	[45]
PVDF solution-cast, thermally poled	-	46.1	Static AFM	[46]
PVDF spin coated, quenched at −20 °C	98	49.6	PFM	[24]
PVDF micropillar, hot pressed, thermally poled	16	64	PFM	[32]
PVDF + 0.4%w.t. rGO in situ, micropillar, thermally pressed	23	66	PFM	[32]
PVDF + 0.1%w.t. rGO in situ, micropillar, thermally pressed	20	75	PFM	[32]

## Data Availability

The data presented in this study are available on request from the corresponding author.
